# 

**Published:** 1899-07

**Authors:** 


### Communicated.

### CIRCULAR LETTER OF THE FOREIGN RELATIONS COMMITTEE OF THE NATIONAL ASSOCIATION OF DENTAL FACULTIES OF AMERICA.

To all who feel any concern in American Educational matters, or in American Professional affairs, the annual meetings to be held at Niagara this summer must prove of the greatest possible interest. It is probable that grave questions, more profoundly affecting the welfare of dentistry, will be discussed and it is hoped settled at that time, than have ever been raised in American dental meetings. The far-reaching subjects that loudly demand consideration concern not America alone, but Europe as well. If dentistry is ever to become a profession in fact as well as in name, if it is ever to occupy the position to which advanced men believe it to be entitled, the professional status and tone in both continents must be brought somewhere near the same level. The future welfare of mankind demands there should be some common understanding of professional affairs.

The first dental school was established in America, and for many years the only institutions for professional training were confined to this country. The Dental Doctors Degree is even now peculiar to American dental schools. For many years, through their excellent practical training, they made American dentistry a synonym for the highest practical efficiency. Then for a time our schools lost ground, and their fair frame became tarnished through the misconduct of some of them, and the criminal laxness of the laws in certain of the states, which permitted the incorporation of fraudulent colleges that sold their doubtful honors abroad and at home, or granted them *in absentia*. It was not until the organization of the National Association of Dental Faculties that any concerted and determined effort to restore the tone of American dental colleges was made, or any practical attempt to bring them to a higher plane, and to force the fraudulent institutions out of existence.

As the natural consequence of the loose methods and legislation of the past, the reputation of the schools that were doing faithful work and maintaining a high standard suffered from the faults of those which were in the habit of receiving unqualified students from abroad, and whose curriculum of study was altogether insufficient. To those not intimately acquainted with American educational matters, there were no means of distinguishing between the good and the bad colleges. All were, by unthinking and uninformed people, charged with the irregularities of the few, and the consequence has been that the reputation of our educational institutions in general has suffered.

Nor was there any complete understanding among the colleges which did desire to maintain a proper standard. Each of our nearly fifty separate states is autonomous so far as education is concerned, that being one of the matters left to domestic regulation by the general government. There can be no compulsory harmony of action, for each college is in a measure a law unto itself, within the limits of state regulation. So long as there was no harmonious concert of procedure, the result of a common agreement and understanding between the different schools whose sole source of income was from the students in attendance, the strife for matriculants and patronage almost necessarily led to a depression of the standard, and too often to irregular graduations.

In the absence of a common law regulating the course of study, some general agreement became a necessity for the maintenance of a proper educational basis. To accomplish this the National Association of Dental Faculties was formed. At the date of its organization the general tone had been so much depressed that it was impossible to establish such a standard for matriculation and graduation as was desirable, but only such colleges were admitted as had the proper facilities for complete instruction, and were conducted by a corps of competent teachers. All other schools were excluded, and their tickets certifying to attendance upon lectures, with their diplomas, were refused recognition by the colleges belonging to the Association. Stringent rules governing attendance, instruction and graduation were adopted, and schools violating them were severely disciplined. The course of study was extended to three full years, and the semesters gradually lengthened until they included from seven to nine months of each year. The curriculum was expanded, until it comprised all the branches of study which the growth of modern professional practice has made necessary. As a consequence, it is believed that each and every one of the colleges embraced in the membership of the National Association of Dental Faculties is now giving thorough professional instruction, and is receiving no students who can not present the evidence of a fair preliminary education. This has been the work of years, for it was impracticable and unwise to make the transition too abrupt. There is much yet to be accomplished, but the Association can point with pride to past achievements, and urge them as a guarantee for its future action.

Two years ago, at the instigation of some of our American graduates abroad, the National Association appointed a standing committee, to be called the Committee of Foreign Relations, whose duty it should be to take into consideration the condition of the American Dental Degree in Europe, and to institute such measures as would prevent the reception of unqualified foreign students by our schools, and to endeavor to give a better understanding of American educational affairs in Europe. It was given authority to appoint European Boards for the purpose of furthering the objects committed to its care, and it was also charged with the attempt to suppress fraudulent and unrecognized American colleges, plenary powers to use Association funds, and even to levy assessments, being bestowed upon it. These extraordinary prerogatives betokened the intense interest which the representatives of the colleges felt in the work. The committee so appointed has labored anxiously and uninterruptedly. It has named the nucleus of a European organization, which it is hoped will be of great benefit to dental educational interests. It has carried on a suit against the most flagrant irregular institution, and has secured a decree condemning it. Before this could be made effective, it become apparent that the repeal of some of the vicious legislation under which incorporation of fraudulent colleges was possible must be secured, and accordingly, in the State of Illinois, bills to accomplish this were introduced, and against strenuous opposition were pushed through the legislature and have become laws. It is believed that if the Committee is sustained by the united voice of the profession its future labors will be more easy, and the entire suppression of all fraudulent schools will be accomplished.

We believe there will be none to dispute the assertion that in the teaching of practical dentistry the dental schools of America have not been excelled by those of any other country. The trouble has been that, for lack of general legislative regulation, the standard of preliminary study has been too low. It is utterly impracticable to raise this to the proper point at one time. Until there shall be a public sentiment created that will sustain effective enactments, it is idle to attempt drastic measures. Such action would only divide the profession and exclude schools which, if the proper time can be given, must of themselves raise their standard to the right level. A regulation that is but a dead letter is far worse than none at all, for it brings law into disrepute. It is utterly hopeless to look for harmony of action through separate state enactments. There must first be an agreement among the representatives of the profession, and then unanimity of action on the part of those of all the states. The attempts at repressive or compulsory action through the different state legislatures as a primary measure, must inevitably result, as it has already done, in a yet greater diversity of laws, and more intense antagonism of professional feeling between different sections. It cannot but end in dividing the profession into two adverse and discordant parties, and the perpetuation of the fraudulent colleges, which it will be impossible to suppress except by unanimity of action. The violent and arbitrary laws already enacted, which encourage and foster bitter animosities, tend to defeat that harmony which alone can bring satisfactory results. If a part of our colleges, existing in the more recently settled and less educationally advanced portions of our common country, are refused recognition and fraternization because they are unable, from lack of time in which to adapt themselves to the changed requirements, to comply with those of a greatly advanced standard, they will thereby be forced into an unprofessional attitude. and will thus perpetrate the existence of irregular American dental schools, to the continued reproach and disgrace of our professional name. We believe it to be far better to advance gradually, but as fast as existing conditions will permit. Hence we deprecate drastic measures, or arbitrary and despotic action. No man or set of men can by independent movements dominate a profession of the dimensions to which dentistry has grown. A proper professional feeling must be a thing for time to bring about. Confidence is said to be a plant of slow growth, and this is eminently true in professional matters.

The wonderful progress made within a few years, under the administration of the National Association of Dental Faculties, leads us to hope that if it is permitted to pursue its own course it will, in a comparatively short time, bring all our colleges up to a point of perfection unattainable by any other means than this mutual agreement and harmony of action. The past is a guarantee for the future, and so long as such rapid progress is being made, it is worse than folly to attempt any violent measures that can be only problematical in their results.

There will be a series of meetings held at Niagara this summer, that can but exercise an over-whelming influence for good or evil on our whole professional future. It is earnestly desired that all who take any interest in our educational affairs will be present at one or more of these meetings. Especially is it important that there be a full consultation between representatives of the colleges and their representative graduates resident in Europe. It is hoped that as many of them as possible will be in attendance, and that so far as is practicable every member of the European Advisory Board will make the pilgrimage to Niagara in July. Nor need the attendance of dentists from abroad be restricted to those thus appointed. The members of the Association will gladly welcome and seek the counsels of any reputable dentist resident in a foreign country.

The meeting of the Foreign Relations Committee will be held at Niagara, commencing on the morning of July 26th. The assembling of the parent body, The National Association of Dental Faculties, will doubtless be called for July 28th, while the National Dental Association, the meeting of the representative men of the profession at large, will convene August 1st. It is desired that foreign representatives in as great numbers as possible will be at Niagara for all these meetings, for while the sessions of the college men have not heretofore been open to strangers, ample opportunity will be given for expression of the views of and consultations with our foreign brethren, and it is within their power to confer lasting benefits upon their profession by making their American confreres fully acquainted with the status of professional affairs abroad.

Respectfully submitted.

W. C. BARRETT,
S. H. GUILFORD,
J. D. PATTERSON,
T. W. BROPHY,
H. W. MORGAN.

Committee on Foreign Relations.

BUFFALO, N. Y., May 20th, 1899.

---

The following is a list of the Dental Colleges of America which at the present time are members of the National Association of Dental Faculties, whose diplomas and tickets alone are recognized and received by the members of the Association.

Alabama—Birmingham Dental College, Birmingham.

California—University of California, Dental Department, San Francisco; Dental Department College Physicians and Surgeons, San Francisco.

Colorado—University of Denver, Dental Department, Denver; Colorado College of Dental Surgery, Denver.

District of Columbia—Columbian University, Dental Department, Washington; Howard University, Dental Department, Washington; National University, Dental Department, Washington.

Georgia—Atlanta Dental College, Atlanta; Southern Medical College, Dental Department, Atlanta.

Illinois—Chicago College of Dental Surgery, Chicago; Northwestern University Dental School, Chicago.

Indiana—Indiana Dental College, Indianapolis.

Iowa—State University of Iowa, Dental Department, Iowa City.

Kentucky—Louisvilie College of Dentistry, Louisville.

Maryland—Baltimore College of Dental Surgery, Baltimore; Baltimore Medical College, Dental Department, Baltimore; University of Maryland, Dental Department, Baltimore.

Massachusetts—Boston Dental College, Boston; Harvard University, Dental Department, Boston.

Michigan—University of Michigan, Dental Department, Ann Arbor; Detroit College of Medicine, Dental Department, Detroit.

Minnesota—University of Minnesota, College of Dentistry, Minneapolis.

Missouri—Kansas City Dental College, Kansas City; Western Dental College, Kansas City; Dental Department Marion-Sims College of Medicine, St. Louis; Missouri Dental College, St. Louis.

Nebraska—University of Omaha, Dental Department, Omaha.

New York—University of Buffalo, Dental Department, Buffalo; New York College of Dentistry, New York; New York Dental School, New York.

Ohio—Cincinnati College of Dental Surgery, Cincinnati; Ohio College of Dental Surgery, Cincinnati; Western Reserve University, Dental Department, Cleveland; Ohio Medical University, Dental Department, Columbus.

Pennsylvania—Pennsylvania College of Dental Surgery, Philadelphia; Philadelphia Dental College, Philadelphia; University of Pennsylvania, Dental Department, Philadelphia; Pittsburg Dental College, Pittsburg.

Tennessee—Tenessee Medical College, Dental Department, Knoxville; School of Dentistry, Central Tennessee College, Knoxville; University of Tennessee, Dental Department, Nashville; Vanderbilt University, Dental Department, Nashville.

Virginia—University College of Medicine, Dental Department, Richmond.

Washington—Tacoma College of Dental Surgery, Tacoma.

Wisconsin—Milwaukee Medical College, Dental Department, Milwaukee.

Canada—Royal College Dental Surgeons of Ontario, Toronto.

The following appointments to membership in the European Advisory Board have been made. The vacancies will be filled at the meeting of the Foreign Relations Committee to be held at Niagara, commencing July 26th, prox.

Great Britain, Wm. Mitchell; Holland and Belgium, J. G. Grevers; Denmark, Norway and Sweded, Elof Forberg; Russia; Germany; Austria and Hungary; Italy and Greece, Albert T. Webb; France, J. H. Spaulding; Spain and Portugal; Switzerland and Turkey, L. C. Bryan.

### SITUATIONS WANTED.

**WANTED**—Situation in Dentistry (Surgical or Mechanical) by English Dental Student of four years practical experience, where opportunities would be afforded of taking post graduate course. Address stating terms etc., to "DENTAL" 117 Chancery Lane, London, England.

### To Readers and Correspondents.

Communications intended for publication, and exchange journals and papers, should be addressed to the Editor of The American Journal of Dental Science, No. 845 N. Eutaw St. Baltimore, Md. All letters relating to the business of the Journal, or containing cash remittances, to be directed to Snowden & Cowman Mfg. Co., 9 W. Fayette Street, Baltimore, Md. Articles for publication should be received by the Editor at least two weeks previous to the first of the month on which the number in which it is designed for them to appear is issued.

☞ The American Journal of Dental Science will contain fifty pages of reading matter in each number, making a volume of about Six Hundred pages,

EXCLUSIVE OF ADVERTISEMENTS.

